# Why won’t it Stick? Positive Psychology and Positive Education

**DOI:** 10.1186/s13612-016-0039-1

**Published:** 2016-02-09

**Authors:** Mathew A. White

**Affiliations:** St Peter’s College, St Peters, Adelaide, SA 5069 Australia; Melbourne Graduate School of Education, The University of Melbourne, Melbourne, Australia

**Keywords:** Education, positive psychology, Positive education, Well-being, Impact, Policy

## Abstract

Following the launch of the positive psychology movement teachers and educators emerged as early adopters of this fledgling science. This approach was called positive education. It describes scientifically validated programs from positive psychology, taught in schools, that have an impact on student well-being. The growing body of evidence about the reach of positive psychology has formed a convincing case to consider well-being an operational goal for educational systems. It is argued that this goal is pivotal and should be pursued in the same way in which we develop strategies to harness academic growth, school retention rates, and student engagement. National education policies can have widespread influence at the grassroots level on school improvement, good quality of classroom teaching and learning, student performance, creating confident and creative individuals and active and informed citizens, but not necessarily on the preventative skills for lifelong well-being. In this article I take stock on the positive education movement. Three approaches to positive education are identified and eight hurdles to the field are noted as reasons why positive education won’t stick in policy. Then, I reflect on two case studies: a Well-being Summit and Round Table held at Wellington College and No. 10 Downing Street and Dr. Martin Seligman’s role as Adelaide’s Thinker in Residence as examples of grass-roots initiatives in well-being. Finally, six strategies are suggested for researchers and practitioners to grow the field. Last, I argued that until research centers focus on the development of common definitions of the key terms underpinning positive psychology, positive education and well-being the impact of the movement will be limited to a handful of institutions as models of best practice.

## Background

Launched at the start of the new millennium, positive psychology was the focus of American Psychologist, edited by Seligman and Csikszentmihalyi (2000a, b). Here, Seligman and Csikszentmihalyi defined positive psychology as the “scientific study of what goes right in life, from birth to death and all stops in between” (Peterson 2006, p. 4). Norrish and Vella-Brodrick (2009) expanded this definition arguing that “positive psychology contributes a comprehensive approach to mental health by adding investigation of positive emotions and human strengths to existing knowledge of mental illness and dysfunction” (p. 275). Huppert (2009) argues that psychological well-being is about lives going well, and it is a combination of feeling good and functioning effectively’ (p. 137). Well-being theory was expanded by Seligman’s (2012) much-discussed multidimensional PERMA model of flourishing, which argues that well-being consists of positive emotions, engagement, relationships, meaning, and accomplishment.

Following Seligman’s call, a movement was established appealing to psychologists to focus on what goes right in people’s lives with the same level of rigour as was applied to anxiety and depression. Researchers, including Rusk and Waters (2013), have demonstrated in their quantitative assessment of the size, reach, impact, and breadth of positive psychology that it “covers many different research topics from a diverse range of disciplines, and that positive psychology literature has been growing rapidly in significance” (p. 207). Since 2000, widespread community-based initiatives and interventions at primary and secondary schools and even colleges and universities have been adopted (Huppert and Cooper 2014).

This article outlines the growth of positive psychology and its application in education and schools, known as positive education. It outlines two case studies: a Well-being Summit and Round Table at Wellington College and No. 10 Downing Street and Dr. Martin Seligman’s role as Adelaide’s Thinker in Residence as examples of grass-roots initiatives in well-being.^1^ It poses a number of hurdles for the growth and sustainability of positive education. Finally, the article explores why well-being won’t stick in policy until researchers and policymakers address these barriers.

### Defining Positive Education

Very early in the launch of the positive psychology movement, applications within educational settings emerged. These approaches were called positive education. Positive education has had various definitions (Seligman et al. 2009; White and Waters 2015; White 2014). O’Shaughnessy and Larson (2014) claim positive education as a paradigm shift in educational approach—where education traditionally focuses on academic accomplishment only. They argue that it is an approach to education and well-being.

I define it as a blend of evidence-based learning from the science of positive psychology and best practices in learning and teaching (White 2014). White and Waters (2015) and White (2014) describe it as an “an umbrella term used to describe empirically validated interventions and programs from positive psychology that have impact on student well-being.” It has an approach characterized as education for both traditional skills and character development (Seligman et al. 2009; Seligman 2013).

White and Murray (2015) have noted, “Positive education programs in schools appear in three forms:

1. Empirically validated and scientifically informed well-being intervention programs that have impact on well-being.
2. Scientifically-informed proactive strategies to the whole school mental health programs in schools.
3. Specific virtues or values and character-based education lessons based in philosophy or values-based learning (p. 14)”.

Evidence to support the impact of positive education is well summarized by Gregory Park in Well-Being and Achievement (2013, p. 7) where he argued, “Educational and psychological research has found similar links between higher student self-control and better life outcomes”. Here is a sampling of recent findings on the benefits of self-control and perseverance:

- Self-control predicted high school grades, absences, and at-home study habits better than IQ (Duckworth and Seligman 2005).
- Self-control predicted homework completion, classroom conduct, and report card grades in a longitudinal study of over 500 middle school students (Duckworth et al. 2012).
- Individuals’ levels of grit—perseverance for long-term goals—predicted several forms of academically-related achievement, including grades at top US universities, retention in elite military academy classes, and ranking in a national spelling competition (Duckworth et al. 2007).
- Changes in a student’s self-control predicted changes his/her school grades over 6 months (Duckworth et al. 2010, 2011).
- Self-control predicts childhood health, too. A study of children progressing into adolescence found that self-control was an important protective factor against becoming overweight (Tsukayama and Duckworth 2010).

However, in schools, there is a demand for positive education programs that, from a critical perspective, could be putting the whole area at risk (White and Waters 2015). At the grassroots level in schools, amongst teachers and educators, there is a thirst for well-being. Within classrooms, each day, teachers are seeing students who are languishing rather than flourishing or, at best, are okay.

The question must be asked: “Why okay?” Australia’s leading voice on preventative approaches to mental health, Professor Patrick McGorry (2014) interviewed by *The Sydney Morning Herald* on 20 August 2014, says, “Mental health … affects four million Australians every year and probably 50 % of us—that’s more like 11 or 12 million—across the course of our lifetime. So it is an absolute sleeping giant. And it is quite extraordinary that this issue is still such a problem. It looked in 2010 as though it was starting to get some real traction with the public, but it actually has died away again.”

### But, Why won’t Positive Education Stick?

Despite evidence of the growth of school-based positive psychology, positive education, and well-being programs across the world, there appears to be a disconnection (Rusk and Waters 2013). It lies between individual schools’ enthusiasm for positive education and integrated reform at a policy level.

Even after 15 years of evidence gathered by researchers in the movement, it seems positive psychology, positive education, and well-being won’t stick to policy. Why is this the case? I argue a number of applied and theoretical hurdles for positive education researchers and practitioners to address. If supporters of positive education hope to gain substantial traction with government policy, researchers should systematically engage with these eight obstacles:

1. *Financial* the view that large sums of money should be spent on training staff in well-being.
2. *It’s a marginal topic* well-being is seen as a distraction from real educational progress in literacy and numeracy.
3. *Either/or thinking* at some policy levels, it is seen as it’s either well-being or another topic.
4. *Maverick providers* providers who deliver questionable training around the topic that claim to have impact with limited evidence.
5. *Scientism* where empiricism is seen as the only way forward and overlooks the philosophical questions which underpin why well-being should be integrated into educational experience.
6. *Not central to good governance* discussions around well-being are not the core business of exceptional governance, which is about developing effective financial and business decision-making models.
7. *The silver bullet* it can be seen to fix all the challenges in education.
8. *Social Economic Status and Culture* it is an excuse not to expect improvement of change in education.

With rising unemployment, global financial instability, and the rise of terrorism, it is no wonder that the discussion about well-being has severe competition in the political arena. For example, in the Australia, schools are called on to engage with and implement The National Assessment Program—Literacy and Numeracy (NAPLAN) testing and the Australian curriculum with its various learning areas in English, mathematics, science, humanities and social sciences, the arts, technologies, health and physical education, languages, and work studies (Australian Curriculum, Assessment and Reporting Authority 2013; Ormerod 2012). Table 1 outlines what I believe are eight reasons why positive psychology and well-being will not stick in a serious policy discussion, and this is what you would hear in the hallway of a school.

**Table 1:** Why won’t it stick? positive psychology and positive education

Reasons against	What is said in the school hallway	What it means
Financial	“We don’t have *any* budget for any of that type of innovation!”	The perspective that a substantial budget is required to drive change and systems improvement
It’s marginal	“You want to focus on well-being? Where are the immediate benefits, what about teaching them to write!”	It is perceived as a *marginal topic* from serious mainstream educational improvement strategies
Either/or thinking	“Well you can’t have your cake and eat it. It is either maths or making them feel good”	At a policy level is seen often seen through the lens of an either/or model: it’s well-being or literacy, well-being or numeracy. It is rarely well-being and numeracy
Maverick providers	“I did the 3 day course let me tell you about my strategy for 1000 students”	Mavericks, swindlers and second tier training stand to make a huge amount of money from well-being training programs under the guide of various institutions promise to ‘train’ teachers in well-being
Scientism	“Well, according to the latest science”	It blindly can become scientism where the scientism method is seen as the most authoritative approach and can over look underlying assumptions and philosophies
Not central to good governance	“Have you any idea what the unemployment rate is our district!”	Discussion about well-being is a distraction from much larger questions policy including: productivity, healthcare and energy
The silver bullet	“You have ticked al the boxes … well, if the well-being of teachers is right, the well-being of students is right—then they will be able to read better”	It can appear be sold as a silver bullet or Trojan Horse that can fix all of the challenges in education. This is sometime characterised by the oversimplified statement “get their well-being right and then everything will follow”
Social economic status and culture	“All are students are languishing, so how can they learn?”	Well-being is an excuse for policy makers not to address declining performance stands in reading, science and mathematics

Nevertheless, not only are there challenges at a policy level, there are questions at a philosophical level. Herein lies the tension of well-being in educational systems. So often, many of the people who are making well-being programs happen in schools do so without recognizing that well-being takes place from within a school, just as well-being takes place within a classroom, within a drama production, and within a sporting team. Each of these groups has values, ways of behaving, and accepted norms. Too many well-being programs are imposed without the care taken to consider existing values within communities before they are integrated.

Kristjánsson (2013a, b) notes there are a number of foundational issues in the growing area of the science of well-being. From his perspective, academics in the area need to grapple with these to create coherent frameworks and a lexicon to be able to breach the divide between discussion about character and preventative models for well-being. So, what are the limitations of the existing system of education?

Darling Hammond (2006) outlines features of traditional school systems, focusing on conformity, or factory-like aspects of systems and behavior. In many instances, schools are structured around organized or suppressed conflict. What do these traditional school systems produce? It seems that many of them produce a raft of reforms that, in some instances, offer a myopic perspective of the world. This appears to be the case, regardless of advances that take place internationally, for example, in Finland.

Until these barriers are addressed, I remain skeptical about whether positive education will ever stick in public policy. Even in the light of the World Health Organization’s prediction that there will be a global crisis in well-being where, by 2030, depression will be the leading burden of disease or that one person commits suicide every 40 s—more than all the yearly victims of wars and natural disasters—it appears policies are reluctant to integrate well-being as a driver between and across community improvement (WHO 2011).

For example, consider the Australian statistics and the apparent lack of impact at a policy level. Since 2012, it has been widely reported that Australia’s Mental Health system is in crisis in particular with “mainstreaming mental health services into the public health sector.” In Australia, suicides have increased by 20 percent over the past 10 years while over the same period; the number of motor vehicle deaths has been reduced by 25 percent to 1200 each year—less than half the number of deaths by suicide. How many suicides does it take until policy makers take note and invest more fully in preventative mental health programs?

The OECD (2015) has recently criticized Australia’s approach to mental ill-health and noted the number of fragmented policies and lack of public spending citing the economic, unemployment, and underperformance issues on Australia’s economy. Australia’s rates of mental illness are widely documented. The rise of the well-being movement in Australia is linked to a greater understanding of preventative approaches to mental health. This year, in the Australian context, this was highlighted by a remarkable weeklong event on Australia’s national broadcaster, raising over $1 million for research. ABC TV and Radio highlighted issues to support Mental Health Week; the ABC went Mental As.

This weeklong event included documentaries, comedy programs, and interviews with leading experts and Australian celebrity ambassadors from music, theatre, sports, and news. While I have outlined many of the initiatives that demonstrate there is evidence to support the claim of an exponential growth about well-being in schools, it can remain marginal in discourse. There is also a widening gap between what many schools claim as well-being in education, why it is important, how they create a conceptual framework to explain this to their communities, and how they operationalize this across the school in interventions, classroom timetables, programs, teacher actions, and behaviors (Huston 2014).

### Growth of Positive Education in Schools

In Seligman and Csikszentmihalyi’s (2000a, b) original paper launching positive psychology, schools were claimed to be an important part of the positive education movement. Seligman and Csikszentmihalyi (2000a, b) defined them as “positive institutions” which are “organizational systems focusing on learning traditional capabilities as well as aiming to move individuals toward better citizenship, responsibility, nurturance, altruism, civility, moderation, tolerance, and work ethic.” It is well recognized that school culture is a key driver in promoting positive attitudes towards learning.

Systematic case studies of schools as positive institutions exploring institutional vision, change management, measurement, and interventions have been limited to a handful of notable studies. These include: Waters, White and Murray (2012), Waters and White (2015 on using appreciative inquiry to support positive change; White and Waters (2015) on examples of the use of the strengths-based approach with students; Kern, Waters, Adler and White (2015) on measuring well-being in students; Kern, Waters, Adler and White (2014) on assessing employee well-being in schools; Alford and White (2015) on positive school psychology; White, Vrodos and McNeil (2015) on the integration of strengths in student leadership; Waters, White, Wang and Murray (2015) on leading whole-school change, and White and Murray (2015) and Waters, White, Wang and Murray (2015) on introducing evidence-based approaches to positive education in schools at a strategic level.

Schools increasingly report playing a role in the management of student well-being as well as the responsibility for teaching critical skills of numeracy and literacy. In the OECD, the average number of hours students will spend in school will be around 6862 h of instruction between the ages of 7 and 14, of which 6710 h are compulsory (OECD 2015, p. 60). In the Australian context, the Queensland, New South Wales and South Australian governments have recognized that well-being and learning are inextricably linked. For example, the Queensland Department of Education and Training’s, Learning and Well-being Framework (2012) claims,

“This ideal learning environment optimizes well-being. It reflects a positive school ethos that makes the school an exciting, stimulating and welcoming place” (2012, p. 3).

Increasingly schools are adopting social and emotional learning programs that promote self-awareness, self-management, social awareness, and social management, for example, the Mind Matters and Kids Matter frameworks and educational programs. Educational policies around student well-being and school culture are key points to creating safe, supportive, and inclusive school cultures that promote learning.

While many school leaders, principals, governing bodies, and teachers agree on the centrality of student well-being, they do not seem to have put it on the agenda of policymakers at the bureaucratic or systems level (Furlong et al. 2014; Pesa.edu.au 2016; White 2013, 2014; Weare and Nind 2014).

### Building Positive Institutions: Top-Down Examples

Following the launch of the positive psychology movement throughout the world, there has been evidence of top-down and bottom-up initiatives in well-being globally that have had an impact on well-being at individual, team, and community levels. Seligman and Csikszentmihalyi (2000a, b) argued that the science has three pillars on which to focus, including the empirical study of positive experiences, understanding positive individual traits, and the building of positive institutions (Peterson 2006).

Seligman and Csikszentmihalyi (2000a, b) believed that the benefits of the application of positive psychology could then be characterized as institutional developments (building new research centers, schools, and universities) and population level (increasing the overall population’s well-being), linked with public policy outcomes (focusing policy discussion on measurement beyond traditional gross domestic product).

I do not intend to provide an exhaustive overview of the past 14 years’ examples of top-down or institutional responses. Positive institutions that focus on moving “individuals toward better citizenship, responsibility, nurturance, altruism, civility, moderation, tolerance, and work ethic,” as Peterson (2006), Seligman and Csikszentmihalyi (2000a, b) characterized, include:

- Increased debate and discussion in public policy around teaching the skills and science of character education in schools in the United Kingdom,
- The establishment of the Jubilee Centre for Character and Virtues at the University of Birmingham,
- and Martin Seligman’s appointment as Thinker in Residence in Adelaide, South Australia.

The Adelaide Thinkers in Residence program was developed in South Australia in 2003 and brought new ideas into the state, translating them into practical solutions to improve the lives of the people who live here under the auspices of the Department of the Premier and Cabinet. These residencies have looked at subjects including health, education, water, technology, climate change, transport, design, and road safety (Seligman 2013). The establishment of the Well-being and Resilience Centre within the South Australian Government led to establishing the South Australian Health and Medical Research Institute (SAHMRI).

Following a recommendation to the South Australian Government, it committed to establishing SAHMRI in 2014 and the endowment and appointment of the chair in positive psychology at the Melbourne Graduate School of Education at the University of Melbourne and the Positive Psychology Centre. The creation of a multi-disciplinary research in positive psychology and education makes a difference in addressing critical educational and psychosocial issues at the Institute for Positive Psychology and Education at the Australian Catholic University.

### Grass-roots Initiatives of Well-being that Stick

On the other hand, there have been some very powerful examples of community engagement related to the topic of well-being that are at the grassroots level in society (Carey 2013). These appear to be democratic in nature, spontaneous, and driven by a group of like-minded individuals who are committed to changes in systems. They also seem to stick, unlike some policy frameworks that do not appear to be enacted. Examples of bottom-up or individual groups, schools, and systems’ responses to well-being include the following: individual schools and systems have started to embrace the social and emotional learning of well-being programs and lessons taught in the United Kingdom and Australia; Welfare to well-being strategies in schools across Australia; Positive Schools conferences in Australia; The creation of the Positive Education Schools Association (PESA) Well-being Roundtable at Wellington College and No. 10 Downing Street, organized by Martin Seligman and James O’Shaughnessy; and the International Positive Education Network (IPEN).

As with all good ideas that start from the ground up, these initiatives vary in size and impact, from not-for-profit organizations running on a shoestring or substantial pro-bono services of a group of committed individuals to large research teams. Each of these initiatives and developments has substantial merit and appears to be united by a similar vision:

- To foster the implementation and development of the science of well-being and its applications in education settings.
- To help individuals and groups or communities to flourish.

Community engagement and policy disengagement despite these initiatives from various directions, the integration of or even serious consideration about well-being by policymakers appears to be lacking. While Kristjánsson (2013a, b) notes that much of this discussion has been characterized in the United Kingdom as “character education,” over the past 5 years, I have observed a more widespread engagement in the topic of well-being.

### Case Study: Positive Education Summit and Round Table—Wellington College UK

In 2013, I attended the Well-being and Positive Education Summit. Organized by Lord O’Shaughnessy, Dr. Martin Seligman, Dr. David Halpern, and Sir Anthony Seldon, the purpose of the summit was to establish the International Positive Education Network (IPEN) and explore developments in well-being and applications in education across the world. Somehow, the summit organizers managed to get into one room with people in well-being from Australia, Mexico, China, Singapore, Europe, and the United Kingdom. This fascinating event was held over 3 days at No. 10 Downing Street and at Wellington College in the United Kingdom.

Sponsors of the event included Professor James Arthur, Professor Kristján Kristjánsson, Ian Morris, and Sir Anthony Seldon. Invited participants included Dr. Ilona Boniwell, Lim Lai Cheng, Dr. Ellen Cole, Professor Angela Duckworth, Dr. Hector Escamilla, Dr. Jane Gillham, Professor Felicia Huppert, Yang Lan, Lord Layard, Steve Leventhal, David Levin, Simon Murray, Dr. Douglas North, Professor Kaiping Peng, and Dominic Randolph.

A report summarizing the Summit was published Graeme Paton, Education Editor on 3 October 2013 in The Telegraph describing the inaugural “Positive Education Summit” as a conference aimed at highlighting the benefits of placing character and well-being on the timetable. Paton wrote, “Dr. Seldon said that character building could be weaved into the mainstream curriculum by giving pupils extra responsibility and offering rewards for those who show responsibility, kindness and other virtues. Separately, schools can also put specific well-being classes on the timetable that show pupils the benefit of exercise, healthy eating, rest, and building strong relationships. International evidence has already shown that schools that have adopted this approach have been able to boost pupils’ academic achievement” (Paton, 2014).

However, I was surprised to learn over the 3 days from colleagues that discussions about well-being and policy are as diverse as the group itself. For example, some discussions focused on the development questions around virtue ethics and “good character” rather than the adoption of a preventative model for mental health. From the outset, we appeared to be speaking a different language. However, all of us were in agreement that we wanted young people to have greater levels of joy, engagement, and meaning, better relationships, and more academic mastery (Huppert 2014). Now, here’s the rub: we are discussing well-being in an “un-well world” (Brasher and Wiseman 2007).

Topics discussed at the Summit included:

- James Arthur and Kristján Kristjánsson outlining research at the Jubilee Centre for Character and Virtues at the University of Birmingham;
- Ian Morris and Anthony Seldon on developments at Wellington College in the UK;
- Ilona Boniwell sharing her experiences from the University of East London and growing application of positive psychology in schools throughout France;
- Lim Lai Cheng on developments of character education in Singapore;
- Angela Duckworth outlining the latest findings of grit and perseverance from her lab at the University of Pennsylvania;
- Hector Escamilla sharing his learning leading the implementation of positive psychology through the systems and educational experience of undergraduate students at la Universidad Tecmilenio;
- Jane Gillham outlining the research on the Penn Resilience Program and related projects teaching resilience competencies in youth;
- Felicia Huppert shared her research from the Well-being Institute and the University of Cambridge and evidence around the impact of mindfulness in schools;
- Yang Lan recounted her experience sharing well-being to millions of people through Chinese Popular Television;
- Lord Layard summarized his significant research from an economic point of view;
- Steve Leventhal outlined the work of the non-profit CorStone working with the poor in India;
- David Levin and Dominic Randolph outlined their joint research from the KIPP Schools in New York and Riverdale Country School, and the early stages of what has since become the Character Lab;
- Simon Murray shared developments at St Peter’s College—Adelaide in Australia;
- Kaiping Peng outlined his substantial research conducted in China and the United States of America.

### Case Study: Dr. Seligman as Adelaide’s Thinker in Residence Limitations of Well-being Policy

In Australia and around the world, rank-and-file interest in well-being is increasing exponentially. This claim is supported by the rapid growth in Australia of teacher associations such as the Positive Education Schools Association and the number of individuals attending free public lectures on the topic at the Sydney Opera House, Melbourne Town Hall, Adelaide Entertainment Centre, and Adelaide Festival Theatre.

Consider the facts from the Seligman Report (2013) regarding voluntary public engagement in Martin Seligman’s lectures as Adelaide’s Thinker in Residence (2012–2014): More than 9000 South Australians attended events and conferences on positive psychology; more than 50 meetings were conducted throughout the residency; more than 25 schools and health settings began active work on positive psychology; 15 partner organizations provided 40 percent of private-sector investments; 9000 people attended public events; 39,500 Twitter accounts were reached during Professor Seligman’s final lecture; and there were more than 27,000 video views on the Adelaide Thinkers-in-Residence YouTube channel, specifically on Seligman material. The Jubilee Centre for Character and Virtues finds that 9 in 10 parents want this type of educational approach (O’Shaughnessy and Larson 2014).

One of the limitations of the wide-scale adoption of well-being in policy is that the majority of character education’s applications are not informed by a scientific knowledge base. This means that there is a lack of theoretical models or frameworks available to inform character education developments. Similarly, teaching, as a profession, still appears to be in search of a practice. This is particularly true when we consider education in contrast to other professions, including law, medicine, accounting, and nursing.

There appears to be a disconnect between grassroots demand for well-being education in schools and evidence-based programs and framework on one hand, and on the other hand, the policy perspectives of various ministers for education. The disconnect between research in well-being, the relationship of well-being and public policy, and grassroots community support highlight this challenge. In Australia, the United States of America, and the United Kingdom, measuring well-being has been a hot topic for the past decade.

This has started to influence the way that public policy is considered from an economic perspective as well as from a psychological perspective (Deeming 2013). The gap between effective measures of the human dimension of work, health, well-being, and education is substantial. Generally, population data have been used for many years in public policy, in a health context. However, the link between the use of population data and the improvement of educational outcomes remains an overlooked area (Collins and Foley 2008).

Martin Seligman’s role as Adelaide’s Thinker in Residence resulted in the development of a strategy for South Australia. Seligman’s role as Thinker in Residence was a remarkable achievement in the development of policy recommendations. This was the first time that Seligman had worked within a political framework, where individuals opted into hear him speak in widely attended public lectures. This report focused on the integration of a whole state well-being strategy for millions of South Australians, making the following recommendations and focusing on three pillars: lead, measure, and build (Seligman 2013).

- *Lead* Position South Australia as a world-leading state of well-being.
- *Measure* Measure the well-being of South Australians.
- *Build* Teach, build and embed well-being science in South Australia.

Why should policy writers care about well-being? In South Australia, there have been various discussions about the potential impact and benefit of Seligman’s report. Seligman’s (2013) report nobly outlined South Australia’s opportunity to create a new vision of the state. While some commentators might sound a note of caution about state-sanctioned well-being programs and measurement, it is worth considering.

Why must well-being be integrated into policy? Just as I argue that there are reasons why well-being will not stick in policy discussion, I assert that there are reasons why it is essential:

The average classroom teacher in Australia will spend up to 10,710 h of class time per year teaching students. In essence, many are acting in loco parentis. In society, we argue that families should be deeply concerned about the well-being of their children. If this is the case, then schools should care about well-being in the same way that parents do. It must be front and center.

There is the unthinking rhetoric of teachers who assert that they are teachers of mathematics first and then do a number of other activities second and third. Well-being is an essential part of the classical vision of education. This is wisdom that comes from the classical view of what it means to live in a civilized world and have a civilizing influence on others. This view of education can co-exist with and be an integral part of academic study.

Therefore, it is fundamental to the concept of an educated individual. As demonstrated by a number of students, schools culture can make an impact on well-being—for good or ill. It is essential that schools actively work to build a culture of well-being that fosters a safe, supportive, and caring environment.

Given that teachers are the central individuals involved in work in schools, anyone who can have that impact in this setting has an obligation to try to make a positive difference in a child’s well-being. Parents, teachers, and children want schools to develop this part of their characters as well as their minds. Improving student well-being improves academic growth (rather than accomplishment), which policymakers and teachers care about.

### A Road Map to Positive Education in Education

The case for well-being in policy, in the years ahead, I believe that if the well-being and positive psychology movement are to be taken seriously within education at a policy level, then centers for the study of positive psychology at the University of Pennsylvania, the University of Melbourne, Australian Catholic University, and others around the world must provide the scientific evidence, organizational benefits, and philosophical arguments to support the integration of well-being in educational policy in the same way that we integrate whole-school approaches to literacy and numeracy.

I believe that this revolves around:

- Having robust evidence-based and scientifically informed answers to critiques;
- not ceding the well-being agenda to the critics;
- the necessity of establishing a compelling case as to why well-being matters in its own right;
- and the need to integrate more coherently an Aristotelian view of the flourishing life, as well as psychologically informed approaches.

The bottom line is that the Aristotelian view is that the flourishing life consists of more than economic outcomes. However, as I write, I hear an element of doubt. How do we really move well-being forward in education? I argue for seven steps researchers and practitioners should address. These are:

1. *Leadership and vision* a unified voice is required across systems and sectors within education to challenge existing views of education as soley focusing on reading, writing and arithmetic.
2. *Governance, strategy and management* As educational systems and individual schools start to grapple with leading the change in the whole school from a positive lens, so too would traditional governance structures evolve. I argue that we will start to see the emergence of positive governance. This is an evidence-based decision-making process that maintains the robust traditions of governance models, including finance and audit, risk management, policies, systems, structures, and strategic frameworks. These have traditionally been deficit-oriented. However, I argue that governance will increasingly start to ask for measures around well-being from a student and staff perspective to respond ethically to employee well-being and to foster positive educational cultures.
3. *Partnerships* schools and systems of education need to focus on developing integrated partnerships with key service providers in mental health, universities and business unified on a vision of well-being for all.
4. *Measurement* A consistent and reliable form of measurement to help define measures of success and also communicate change and continuous improvement.
5. *Knowledge transfer* Develop an approach to the introduction, management of change and improvement agenda that clearly articulates examples of roles and responsibilities that help to drive well-being improvement across the whole system.
6. *Interventions* Deeper collaboration between and across schools of what interventions have evidence-based outcomes of impact is needed. Significant, interventions that can be aligned with school policies, practices and behavioral expectations will foster the growth of positive education across educational systems.
7. *Communications* clear communications that are able to demonstrate the benefits of positive psychology, positive education and well-being more broadly within education.

It will be important to clearly demonstrate the evidence of impact on objective and subjective measures of social and emotional well-being on individuals’ and groups’ sense of self. We need to overcome the ideological, class, and cultural barriers that serve as underlying assumptions in politics in the rhetoric of well-being. We must highlight the work, case studies, and research of key people who are not mavericks! We must provide case studies of the best practice that outline that it is possible to teach both well-being and academic accomplishment.

Can the thinking that caused the problem fix it? Isn’t history just one damned thing after another? Having directed a number of large well-being projects in schools, I am often asked, “How do you do it?” I believe that, at an important level, it lies at the heart of leadership, the process for problem solving and systems improvement. I now feel that, to solve contemporary problems around well-being, it is important to understand the systems that created it, but not to be bound by the thinking that created the issues in the first place.

In schools, what we do today matters for the world our students will enter tomorrow. In 2016, current year 2 students will enter it when they leave school in 2030. I have summarized in Table 2: Steps towards implementing well-being policy in schools, what I believe is essential to equip these year 2 students effectively with the skills to navigate this pathway. These key drivers in the discussion are in the introduction of well-being science into pre-service teacher training and education, professional development for existing educators in character and well-being science, development of professional leadership and well-being science, and the evolution of school governance to focus on the well-being of schools (White 2014).

**Table 2:** Steps towards implementing well-being policy in schools

Steps	Commentary
1: Leadership and vision	For well-being to be taken seriously it required committed and clear leadership with broad vision and mission to move schools/educational settings to move from being good, great to excellent
2: Governance, strategy and management	Clear alignment between the roles and responsibilities of governance, management and strategy development and the operational steps that are required to ensure that these improvements are sustainable and have owners to make it happen
3: Partnerships	Mutual partnerships with external thought leaders and experts in the field to build internal capability
4: Measurement	Rigorous measurement tools to ensure that leaders are able to articulate measures of success and key moments during project delivery
5: Knowledge transfer	Models that are developed to ensure that roles and responsibilities in schools a clear and cohesive definitions around key terms that are aligned across the whole system
6: Interventions	Evidence-based programs that have been shown to have positive impact on student well-being and development when fidelity to the course is observed
7: Communications	Clear and coherent communications that demonstrate the goals, objectives and strategies for well-being

## Conclusions and Further Questions

Why won’t positive psychology, positive education, well-being, and public policy stick? Despite the genuine growth and interest in well-being, I remain skeptical about well-being’s ability to stick in public policy. As McDaid (2014) and O’Donnell (2014) note, until economists, school leaders, and researchers collaborate to create a consistent theoretical framework to guide policymakers to appreciate well-being’s pivotal role in educational accomplishment, I believe the positive education movement is at risk of either not being taken seriously, being dismissed or being limited to a handful of extraordinary examples in schools acting as centers of excellence across the world.

It is my contention that researchers and practitioners in the positive psychology and positive education movements start to collaborate with the theoretical hurdles and reasons why well-being will not stick in policies to enable sustainable change and to bolster the next 15 years of research. Until researchers and practitioners address these fundamental hurdles the sustainability and impact of the positive education movement with education more broadly will be diminished. Research centers must focus on the development of common definitions of the key terms well-being, positive psychology and positive education; recommendations for how to approach whole systems change improvement; articulating the social and financial return on investment for such an approach; and developing tools to help manage and drive sustainable change.

Government policies should strive for individuals and communities to develop the skills required to move whole communities towards flourishing. This evolution will enable individuals who are experiencing moderate levels of mental health to progress towards a flourishing state.

## Footnotes

1 The Positive Education Summit 2–5 October 2013 held at Wellington College and No. 10 Downing Street in the UK influenced this article. I acknowledge thought provoking discussions with Professor James Arthur, Dr. Ilona Boniwell, Professor Angela Duckworth, Dr. Jane Gillham, Professor Felicia Huppert, Professor Kristján Kristjánsson, Lord O’Shaughnessy, Dr. Héctor Mauricio Escamilla Santana, Sir Anthony Seldon, Professor Martin Seligman and Professor Kaiping Peng and during the Summit.
